# P-1824. Implementing Hepatitis C Treatment During Inpatient Admissions at a US-Based Tertiary Hospital

**DOI:** 10.1093/ofid/ofaf695.1993

**Published:** 2026-01-11

**Authors:** Anna C Scialli, Tara Vijayan

**Affiliations:** David Geffen School of Medicine, University of California Los Angeles, Los Angeles, CA; University of California Los Angeles, David Geffen School of Medicine, Los Angeles, CA

## Abstract

**Background:**

Curative and well-tolerated direct-acting antivirals (DAAs) for hepatitis C virus (HCV) infections have been available for over a decade yet lack of access persists for many patients with HCV, particularly among individuals who are chronically unengaged in primary care. We have developed a quality improvement protocol to decentralize HCV treatment by enabling hospitalists to initiate care in the inpatient setting.

**Figure:**
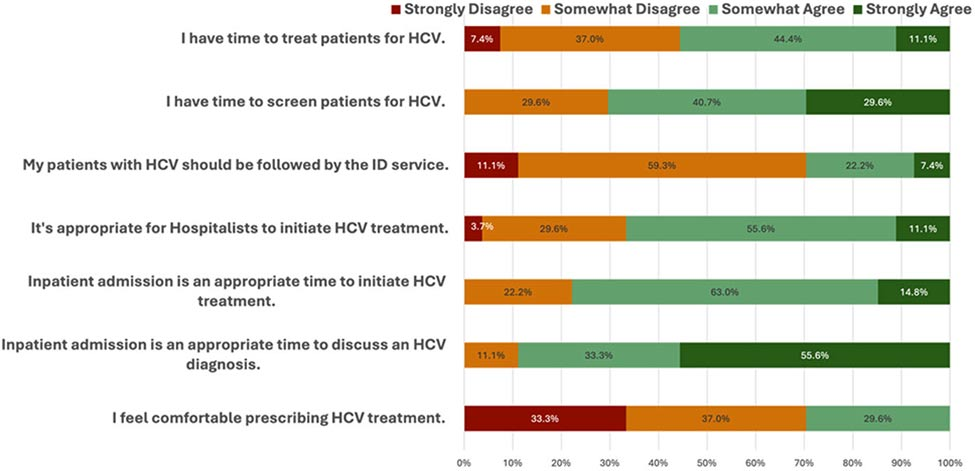
Hospitalist survey responses: attitudes towards inpatient treatment of HCV, September 2024, N=27

**Figure:**
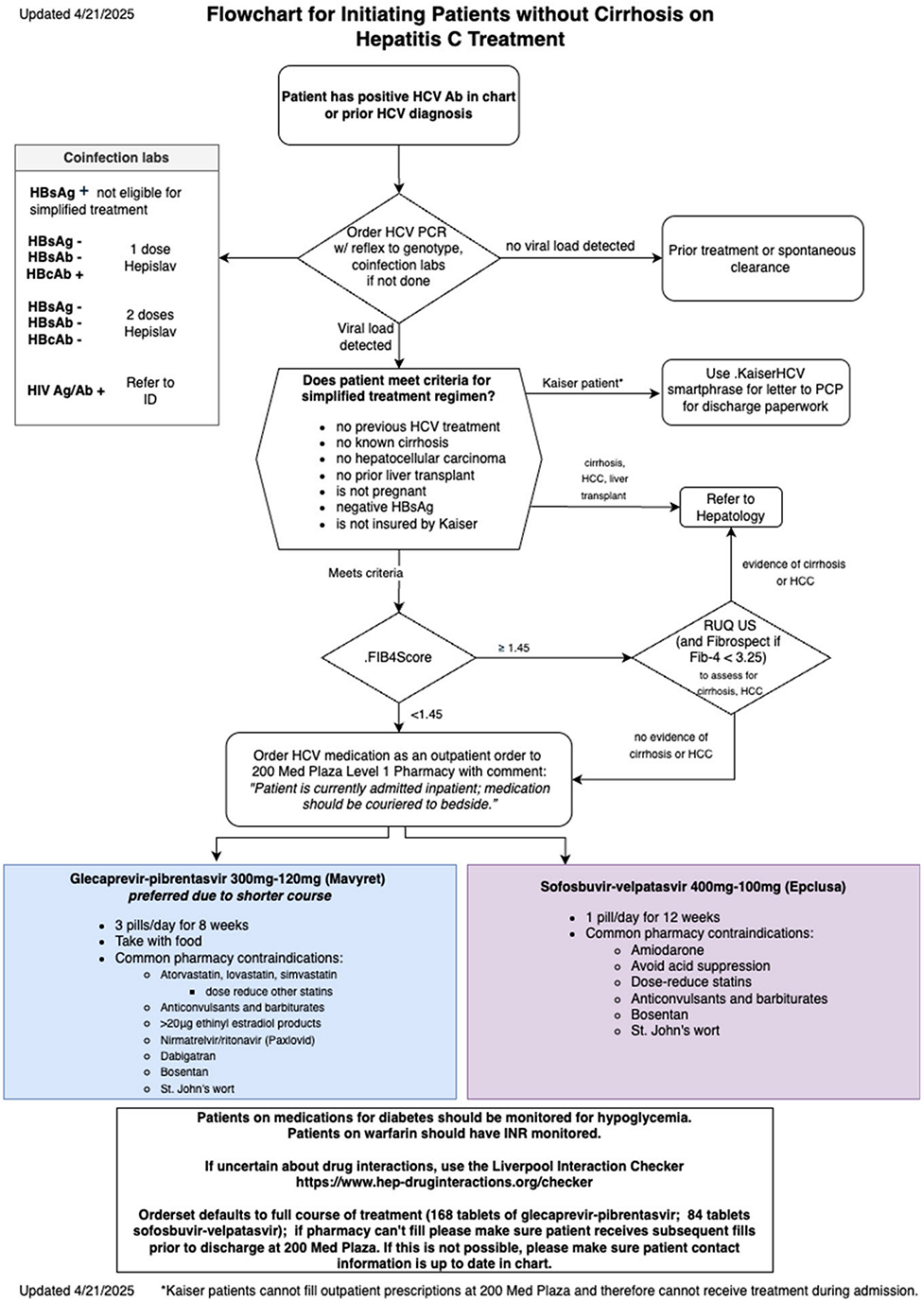
Treatment algorithm for simplified HCV treatment on the inpatient medicine service.

**Methods:**

A survey was distributed to all hospitalists within our tertiary medical center to assess knowledge of and willingness to treat HCV in the inpatient setting, as well as perceived barriers to initiating treatment. Based on hospitalists’ reported needs, order sets were built within the electronic medical record (EMR) to help guide treatment decisions. We describe four patients who were reviewed for treatment from February to April 2025.

**Figure:**
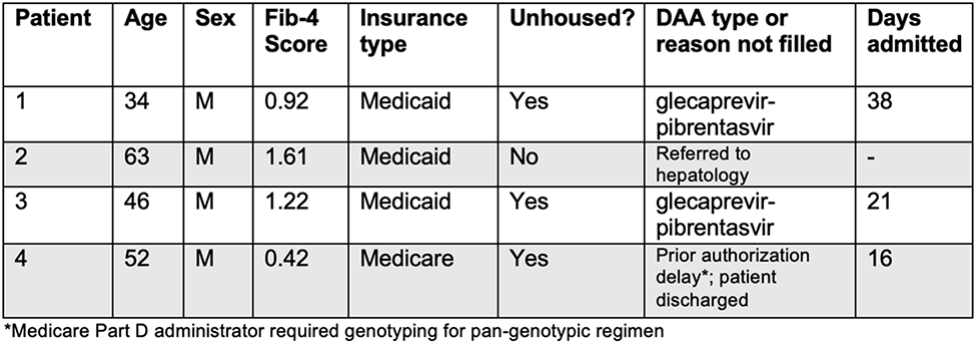
Characteristics of patients eligible for inpatient DAA treatment using the QI protocol as of April 30, 2025.

**Results:**

The majority of respondents among internal medicine hospitalists felt favorably about initiating HCV treatment, however, were unsure of how to do so (Figure 1). A treatment flowchart was provided to guide hospitalists through determining patient eligibility for simplified treatment (Figure 2). Given how cost prohibitive it is to keep DAAs on hospital formularies, we implemented a process to order the antivirals at our institution’s specialty pharmacy and had the medications delivered to the beside. Two of the four patients received treatment while admitted and both were unhoused.

**Conclusion:**

Implementing EMR interventions such as order sets and leveraging the institution’s specialty pharmacy can facilitate the initiation of HCV treatment in the inpatient setting. This intervention is critical to providing access to patients underserved in traditional, outpatient healthcare settings, such as individuals who are unhoused and individuals who use drugs. Our protocol provides a roadmap for other health systems to facilitate the treatment of HCV by internal medicine hospitalists.

**Disclosures:**

All Authors: No reported disclosures

